# Waterproofing Spray-Associated Lung Injury Review: Differences between Cases of Early and Delayed Improvement of Waterproofing Spray-Associated Lung Injury

**DOI:** 10.3390/jcm12062404

**Published:** 2023-03-21

**Authors:** Norio Kodaka, Chihiro Nakano, Takeshi Oshio, Kayo Watanabe, Kumiko Niitsuma, Chisato Imaizumi, Takatomo Hirouchi, Yuto Yoshida, Yuka Yamada, Hiroto Matsuse

**Affiliations:** Division of Respiratory Medicine, Department of Internal Medicine, Toho University Ohashi Medical Center, 2-22-36 Ohashi, Meguro-ku, Tokyo 153-8515, Japan

**Keywords:** bronchoalveolar lavage fluid, Krebs von den Lungen-6, leukocytosis, waterproofing spray

## Abstract

Waterproofing spray-associated lung injury (WALI) is an acute respiratory disorder characterized by bilateral diffuse lung injury on chest computed tomography (CT). In most cases, the symptoms and abnormal radiographic findings of WALI patients improve spontaneously over several days; however, some cases have persistent symptoms and abnormal shadows for >1 week. The distinctive features of each WALI are unknown. Herein, we present two new cases of WALI that we encountered in our hospital, and we previously reported two other cases of WALI. We examined the characteristics of WALI in our cases and 39 other cases of WALI definitively diagnosed and reported in PubMed and the Japan medical board with verifiable data during a 15-year period. We compared the clinical characteristics of the 22 cases in which the patients’ symptoms were resolved within 1 week (early improvement) to those of the 21 cases in which the symptoms were resolved after >1 week (delayed improvement). The WALI cases with delayed improvement had significantly more shadowing that extended over the entire lung field and was not biased in intensity toward the upper or lower lung field. In addition, the serum white blood cell (WBC) counts and serum Krebs von den Lungen-6 (KL-6) levels differed significantly between the cases of early and delayed improvement of WALI.

## 1. Introduction

Waterproofing sprays are readily available products that can be used on coats, shoes, leather, and solids to ensure water and dirt resistance at home. These products are widely used because of their convenience. Typically, waterproofing spray-associated lung injury (WALI) is more likely to occur with the high rate of use of sprays and in smokers. In addition, typical cases present with respiratory symptoms such as cough and rhinorrhea, and other features such as dyspnea, fever, leukocytosis, elevated C-reactive proteins (CRP), and hypoxemia a few hours after the use of the waterproofing spray. These cases also often show a pulmonary edema-like shadow on imaging [1,2]. Most cases completely recover within 1 week with symptomatic treatment such as oxygen inhalation, and their prognosis is considered to be good. However, there are some reports of prolonged symptoms of WALI.

Waterproofing sprays are generally composed of volatile solvents (such as isopropanol or xylene), water-repellent agents (such as fluorinated resins), and spray agents (such as butane or compressed air) [3]. The inhalation of fluorinated compound resins (used as water repellents) is known to cause acute lung injury [4].

This is because the inhalation of water-repellent agents such as fluorinated resins can cause lung damage. In a previous report, the mechanism underlying WALI, namely, the inhalation of fluorinated resins in a waterproof spray, induced macrophage infiltration and alveolar septal thickening, increased airway resistance and reduced the expiratory flow rate in mice [5,6]. However, the clinical characteristics of this pulmonary damage, which may be early or delayed, remain unclear.

There are few reports on WALI, and whether its improvement is early or delayed has not been investigated; therefore, we reviewed the reports of previous studies, in addition to the two new cases we encountered to identify the differences in the characteristics between the cases of early and delayed improvement of WALI.

## 2. Case Reports

### 2.1. Case 1

A 42-year-old man who sprayed his shoes with waterproofing sprays in the bathroom sequentially smoked a cigarette; after that, he had a sore throat and began coughing, with no improvement in his symptoms for 2 weeks. Thus, he visited our hospital.

**Figure 1:**
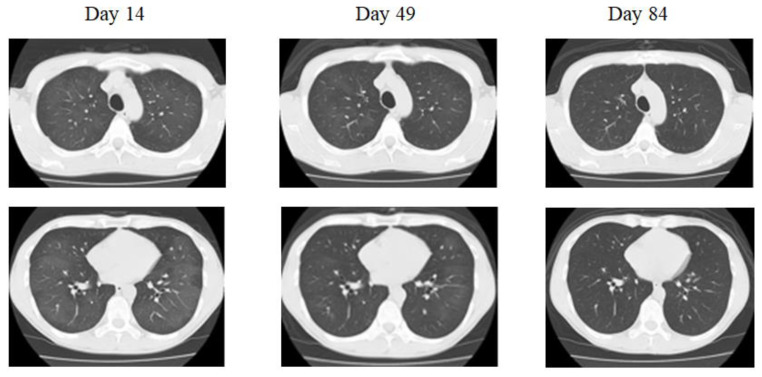
Chest X-ray and chest computed tomography (CT) in case 1. Bilateral diffuse, ground-glass opacity can be observed, but no pleural nadir shadow. A mosaic pattern can be observed in some areas (Day 14). There is a trend toward shadow reduction in both the upper and lower lung fields, but shadows are still present (Day 49). Improved completely (Day 84).

The initial findings were as follows: blood pressure: 125/83 mm Hg, pulse: 90 beats/min, body temperature: 36.1 °C, oxygen saturation (SpO_2_): 97% in room air, no cyanosis, clear respiratory sounds, and no obvious dry or moist rales, heart murmur, or edema of the extremities. Blood analyses conducted upon admission revealed that the patient’s complete blood counts, liver function, renal function, electrolytes, and CRP levels were within normal limits, except for the elevated levels of lactate dehydrogenase (LDH; 667 IU/L, normal range: 275–512 IU/L). The serum Krebs von den Lungen (KL)-6 level was 390 U/mL (normal range: <500 U/mL). The percentage of macrophages in bronchoalveolar lavage fluid (BALF) (Day 21) was 97.0%. Chest X-ray and chest computed tomography (CT) performed upon arrival revealed that diffuse ground-glass appearances in both lung fields and peripheral areas were absent (Figure 1).

He had a 28-pack-year history of cigarette smoking for 28 years. He did not take steroids. The light cough persisted and the chest CT performed approximately two months after the onset revealed a residual ground-glass appearance (Figure 1). Finally, three months later, the ground-glass appearance had cleared on the chest CT (Figure 1).

### 2.2. Case 2

A 40-year-old woman who worked as a dressmaker presented with dyspnea and a cough immediately after using large amounts of a waterproofing spray. After the onset of symptoms, the patient was urgently hospitalized at night. The initial findings were as follows: blood pressure: 118/54 mm Hg, pulse: 119 beats/min, body temperature: 36.8 °C, oxygen saturation (SpO_2_): 95% in room air, no cyanosis, with fine crackles, and no obvious dry or moist rales, heart murmur, or edema of the extremities.

**Figure 2:**
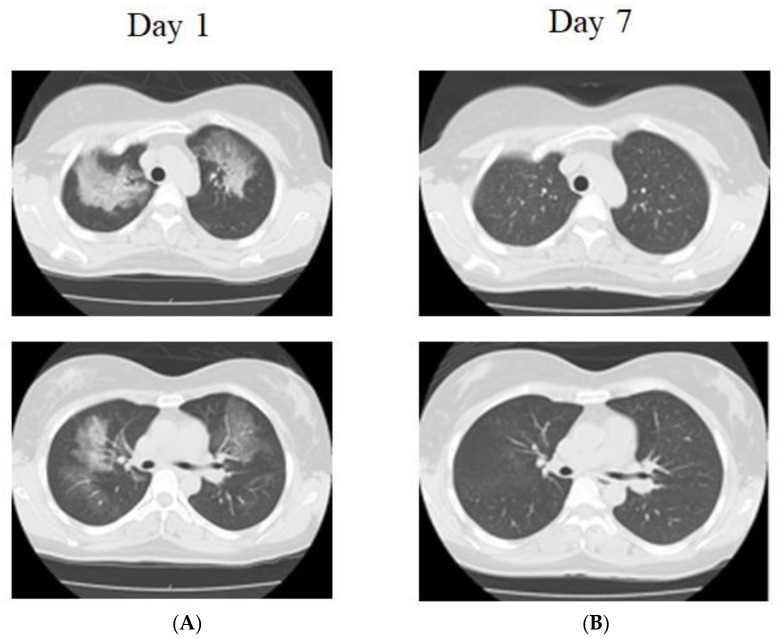
Chest X-ray and chest computed tomography (CT) in case 2. (**A**) Bilateral diffuse, ground-glass opacity predominates in the upper lung fields, with a partially mosaic pattern, and no pleural nadir shadow. There is no pleural effusion (Day 1). (**B**) Improved completely (Day 7).

The CT scan revealed that frosted shadows, mainly in both the upper lung fields and peripheral areas, were absent (Figure 2). She had a 5-pack-year history of cigarette smoking for 20 years. The laboratory data revealed leukocytosis (16,900/μL) with neutrophilia (93.5%). The C-reactive protein level was 0.61 mg/dl, and the serum lactate dehydrogenase concentration level rose to 398 IU/L (normal range: 275–512 IU/L). The serum Krebs von den Lungen (KL)-6 level was 239 U/mL (normal range: <500 U/mL). The percentage of macrophages in BALF (Day 5) was 96%. She did not take steroids. One week later, the shadows improved on the CT scan (Figure 2). Two patients were suspected to have WALI based on the radiological findings and anamnesis. However, other diseases, such as infection and eosinophilic pneumonia, were also considered and bronchoscopy was performed and the possibility of other diseases was ruled out.

## 3. Methods

### 3.1. Study Design

**Figure 3:**
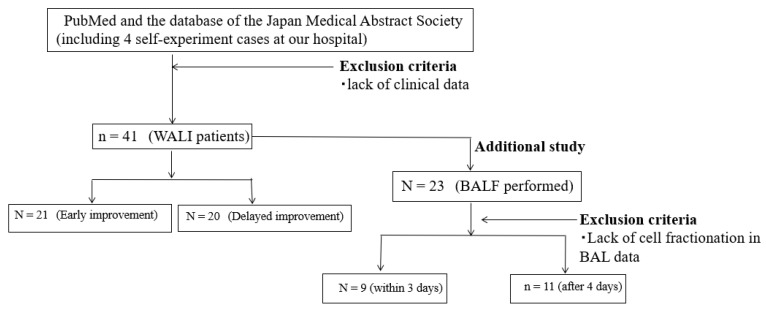
Flow chart of patients diagnosed with WALI between April 2007 to April 2007 to March 2022 of PubMed and the database of the Japan Medical Abstract Society (including 4 self-experiment cases at our hospital). WALI = Waterproofing spray-associated lung injury.

Previous case reports of WALI from PubMed and the Japan Medical Abstracts Society database were collected from April 2007 to March 2022, using the keywords “waterproofing spray,” “water repellent,” “fluoropolymer,” and “chemical pneumonitis” in English. Four patients were hospitalized for WALI at our institute (2 case reports have already been published in Japanese) [7,8]. The combined data of those patients and our patients were reviewed (Figure 3). Patients with chemical pneumonitis due to the inhalation of products other than waterproofing sprays were excluded, and the articles that did not report the duration of improvement and laboratory data were excluded. The age, sex, pack-years, chief complaints, radiological findings, use of steroid treatment (or not), and duration of WALI improvement were investigated [2,7,8,9,10,11,12,13,14,15,16,17,18,19,20,21,22,23,24,25,26,27,28,29,30,31,32,33,34,35,36,37,38,39,40,41].

This study compared the characteristics of patients whose symptoms and abnormal shadows were resolved within 1 week to those whose symptoms and abnormal shadows were resolved after >1 week (Table 1).

### 3.2. Time-Lapse to Improvement

In this study, the time required for WALI improvement was defined as the period between when the symptoms and radiological findings were noted and when they improved. However, for case reports in which the number of radiological findings was small, the time to symptom improvement was given priority (for example, a case in which the symptoms improved in 5 days but the CT scan revealed a persistent shadow 2 weeks later was considered to require >2 weeks to improve, whereas in a case in which the symptoms improved in 5 days, the patient was discharged, and the CT scan performed 2 weeks later revealed improvement was considered to require 5 days to improve).

### 3.3. Assessment of Radiological Findings

We referred to the radiological findings and comments in the respective case reports to determine whether there was evidence of minimal change in all lung fields or whether there was an enhancement bias of the shadows in the upper or lower lung fields.

### 3.4. Additional Study: BALF

The study was approved by the Ethics Committee of Toho University Ohashi Medical Center, an ethics committee that reviews research on human subjects (approval no. H 22063). This board makes decisions as per the principles of the Declaration of Helsinki.

All data were analyzed and processed using Prism (version 9). Student’s t-test, the Mann–Whitney U test, and Fisher’s exact test were used to compare the groups to each other. The threshold of statistical significance was set at *p* = 0.05 (two-tailed).

## 4. Results

Of the cases reported here, we reviewed 43 cases of sprayed lungs, including 2 cases previously reported at our hospital, and a search of the existing literature over the 15-year period from April 2007 to March 2022. In most of the cases, fluorinated resins were used, and in only three cases were these resins not used. Smokers accounted for >90% of the patients involved.

There were 22 cases in which symptoms improved within 1 week and 21 cases in which the symptoms persisted for >1 week (Table 1).

Compared to the patients with early improvement, those with delayed improvement had significantly higher serum KL-6 levels and significantly lower WBC counts.

Compared to the patients with early improvement, those with delayed improvement had entire lung fields of abnormal shadows, which were significantly not biased in intensity toward the upper or lower lung fields. No significant differences were found in the BALF cell fractions between the two groups.

In an additional study, BALF was performed in 23 of 41 cases of WALI. Regarding the timing of the implementation of BALF, it was performed within 3 days in 10 cases and after 4 days in 13 cases (Table 2). A comparison of these two groups showed that those in whom BALF was performed within 3 days had more neutrophils in the BALF fraction, whereas those in whom BALF was performed after >4 days had fewer neutrophils and more macrophages (Table 2).

## 5. Discussion

The patient in case 1 had prolonged symptoms for >1 week, whereas the symptoms of the patient in case 2 improved within 1 week. The patient in case 1 smoked more than the patient in case 2, and the former had more widespread lung shadows than the latter; however, the latter was more dyspneic than the former. 

The patients in both cases in this study improved spontaneously without oxygen or steroid therapy; however, there was a significant difference in the time required for improvement between case 1 and case 2. The causes of this difference are discussed separately as follows.

### 5.1. Smoking

Previous reports have shown that WALI can occur even with only water-repellent agents such as fluoropolymers; however, heating substances (heated by smoking or other means) are said to be an aggravating factor in sprayed lungs [33,42]. In patients with preexisting lung diseases such as emphysema, prolonged and/or severe lung damage caused by waterproofing sprays has been reported [10]. Considering that these reports are also available, it could be that a more prolonged history of smoking might predispose patients to prolonged lung damage. Kondo et al. also point out that a patient’s smoking habit is an exacerbating factor in smoking-induced chronic lung damage, as the range of respiratory impairment is extensive [2]. However, the present comparisons of early-improvement cases versus delayed-improvement cases did not reveal significant differences.

Yoshizumi et al. also reported that among the 13 cases of lung injury due to the inhalation of waterproofing sprays, the time to recovery was 4–60 days in 13 cases in which the patient smoked after using a waterproofing spray, whereas it was 7–11 days in 3 cases in which the patient did not smoke after using the spray. We speculate that the presence or absence of smoking after the inhalation of the waterproofing spray might have affected the severity of the disease [9]. Although smoking immediately after inhalation was not one of our evaluation items because it was not described in many case reports, smoking immediately after inhalation was confirmed in 13 cases of delayed improvement and 8 cases of early improvement.

### 5.2. Serum WBC Fractionation

The cases with early improvement had significantly higher WBC counts than those with delayed improvement. However, the significant difference in the WBC count between the early-improvement cases and delayed-improvement cases could be because all of the early-improvement cases were reported within 3 days of the onset of symptoms, whereas 7/21 of the delayed-improvement cases were reported >4 days after the onset of symptoms.

The median WBC count of blood drawn within 3 days was 17,200 (11,700–23,200), and most cases reported within 3 days of the onset had high WBC counts.

Although the exact cause of leukocytosis is unknown, it could be due to the rapid lung injury that occurs throughout both lung fields. An abnormally high WBC count early in the course of the disease may help in the diagnosis of WALI.

### 5.3. Serum KL-6, SP-D

Serum KL-6 is a mucinous high-molecular-weight glycoprotein classified as human MUC1 mucin, which has been reported to serve as a sensitive marker for interstitial lung diseases [43,44]. Therefore, serum KL-6 levels are elevated when the alveolar epithelial layer is damaged by interstitial pneumonia or other causes. In our study, the serum KL-6 levels of patients with WALI were also within the normal range. This might be because WALI does not strongly induce lung fibrosis [7].

However, for reasons that are unknown, the delayed-improvement cases had significantly higher values than the early-improvement cases. SP-D levels were within the normal range and did not differ significantly between the early-improvement cases and delayed-improvement cases.

### 5.4. Steroids

In the present study, there was no significant difference in the use of steroids between the early-improvement cases and delayed-improvement cases. However, more cases were treated using steroids in the delayed-improvement group than in the early-improvement group (Table 1). Since the results do not indicate that steroids themselves are effective against WALI, it was expected that many of the patients had been using steroids because of their prolonged symptoms.

In fact, there is no unanimity of opinion regarding the use of steroids for lung damage caused by waterproofing spray inhalation. Shimizu et al. suggest that the use of steroids might be considered in cases in which there is an underlying pulmonary disease (such as COPD) or in cases in which the respiratory condition does not improve even after several days of inhalation [10]. According to Sanno et al., the efficacy of steroids is determined by the possible involvement of immunological mechanisms other than cytotoxicity as the cause of prolonged WALI [7]. However, there are currently no published studies on this subject, and we are skeptical about steroid use.

### 5.5. Radiological Findings

Generally, the CT images of WALI were similar to those of pulmonary congestion and pneumocystis pneumonia, with the region just below the pleura being spared in most cases, which explains why WALI is characterized by a large spray component, with the peripheral areas on CT components (just below the pleura) often missing.

In the present study, only a single case had unilateral shadowing. Some cases had more intense shadows in the upper lung fields or in the lower lung fields. Patients with delayed improvement in WALI were significantly more likely to have a diffuse, all-pulmonary-field shadow that is not biased in intensity toward the upper or lower lung fields for unknown reasons.

Sawamoto et al. reported that it took 130 days for WALI to improve and speculated that emphysematization on CT may be the cause of the prolongation [11]. In the present study, most of the patients were smokers; however, only a few case reports describe emphysematization on CT images. Therefore, it was difficult to make a judgment on this point.

### 5.6. BALF WBC Fractionation

In the study, among the WALI patients, BALF was performed in 8 early-improvement cases and 13 delayed-improvement cases in which the leukocyte fractions can be described; however, there were no significant differences in these fractions. Regarding this result, we thought that the dates on which BALF was performed from the onset of WALI were not constant, which could affect the validity of these comparisons (Table 1).

Since only a few reports on BALF for WALI have been reviewed, an additional BALF study was conducted. In this additional study, we compared the WBC fraction in the BALF between patients who underwent early BALF and those who underwent late BALF. BALF was performed in 23 of 43 cases. Among them, three cases without detailed cell fractionation data in BALF were excluded (Figure 1). BALF was performed within 3 days in nine cases. BALF was performed after 4 days in 11 cases (Table 2). A comparison of these two groups revealed that those within 3 days had significantly more neutrophils in the BALF fraction than those who had undergone BALF after >4 days, as the latter group had significantly fewer neutrophils, mostly occupied by macrophages (Table 2).

Although during the early stages of WALI, it can be difficult to perform BALF due to dyspnea, it is also meaningful to confirm an increase in the number of macrophages after >4 days for the diagnosis of WALI. The fact that most of the macrophages are present in the lung fields (despite the presence of intense shadows in the lung fields) is a characteristic finding of WALI. An increase in the number of macrophages after Day 4 of the onset of BALF is characteristic and useful in the diagnosis of WALI, in addition to medical questionnaires. In this regard, Ota et al. performed BALF twice on the same patient and found that the neutrophil percentage increased to 42.3% in BALF performed on Day 2 of the disease onset; however, on Day 11 of the disease onset, without treatment, the neutrophil percentage decreased to 1.9%, suggesting that neutrophils increase in number only in the early stage. Shimizu et al. also speculated that neutrophils are involved in the pathogenesis of early lung injury due to waterproofing spray inhalation [10].

### 5.7. Limitations

This study is an aggregation of studies published in English and Japanese only. The number of cases of WALI is small, and many cases are not reported. It is expected that many cases of early improvement were not reported or improved without the patient going to the hospital. Thus, early-improvement cases outnumber the delayed-improvement cases. 

As for the radiological findings, we made our judgment based on the comments and imaging findings in the respective articles. However, it was not possible to objectively score the shadows because not all the CT slices were not presented. The content of WALI has been under investigation for 15 years, and the components of the sprays are not exactly the same. However, they almost always contain fluorine, which is a water repellent as far as we know.

## 6. Conclusions

Regarding the early improvement and delayed improvement of WALI, patients with diffuse spread of the lung injury to all lung fields without biased intensities were significantly more prevalent in the delayed-improvement group. Additionally, the WBC counts and serum KL-6 levels differed significantly between the early-improvement and delayed-improvement cases of WALI.

In an additional study, it was observed that increased macrophage counts in BALF findings after Day 4 are characteristic of WALI.

## Figures and Tables

**Table 1:** Comparison of clinical characteristics of early and delayed improvement of WALI (n = 43).

Characteristics	Early Improvement n = 22	Delay Improvement n = 21	*p*-Value
Age, median (IQR), year	42 (36–50)	45 (33–58.5)	0.46
Sex (male/female)	16/6	19/2	0.24
Smoking history (current/former/never/unknown)	20/1/0/1	17/1/3/0	0.34
Pack year ^a^	22.5 (5–35.25)	30 (10–44)	0.17
Symptoms			
Fever, n (%)	14/20 (70.0)	15/21 (71.4)	1.00
Dyspnea, n (%)	18/22 (81.8)	19/21 (90.5)	0.66
Cracles, n (%)	4/20 (20.0)	6/21 (28.6)	0.72
Hypoxemia	12/21 (57.1)	7/21 (33.3)	0.21
Radiological findings			
Bilateral/unilateral	21/1	21/0	1.00
Consistent shadow/biased shadow/unknown	7/14/1	15/6	0.03 *
Systemic steroid therapy, n (%)	9/22 (40.9)	15/21 (71.4)	0.07
WBC (/μL) ^b^	17,250(13,030–24,918)	103,00 (7345–19,550)	0.009 *
CRP (mg/dL) ^c^	6.44 (2.29–10.1)	3.73 (0.89–15.29)	0.66
KL-6 (U/mL) ^d^	233 (161–296)	378 (206–389)	0.03 *
SP-D (ng/mL) ^e^	96.7 (18.8–111)	121 (84.95–206.8)	0.11
BALF cell fractionation			
TCC (10^5^/mL) ^f^	2.65 (1.19–23.5)	6.15 (0.68–8.5)	0.64
Neuro (%) ^g^	44.5 (4.13–76.6)	8 (0.55–34)	0.12
Lympho (%) ^g^	1.8 (0.8–4.5)	3 (2.5–5)	0.12
Eosino (%) ^h^	0.1 (0–0.55)	3 (0.1–7)	0.15
Macrophage (%) ^g^	47.5 (22.5–91.3)	85 (56–92.6)	0.26

Data are expressed as medians (interquartile range) or numbers. ALB, albumin; BMI, body mass index; CRP, C-reactive protein; TP, total protein. ^a^: Early n = 18, delayed n = 18; ^b^: early n = 20, delayed n = 18; ^c^: early n = 18, delayed n = 17; ^d^: early n = 11, delayed n = 12; ^e^: early n = 9, delayed n = 9; ^f^: early n = 8, delayed n = 12; ^g^: early n = 8, delayed n = 13; ^h^: early n = 8, delayed n = 11. * *p* < 0.05.

**Table 2:** Comparison of BALF within 3 days of waterproof spray inhalation and after 4 days.

Characteristics	Within 3 Days n = 9	After 4 Days n = 11	*p*-Value
TCC (10^5^/mL) ^a^	6.7 (1.19–23.5)	2.99 (0.6–8.5)	38.4
Neutro (%) ^b^	59 (32.5–80.4)	2 (0.5–7.5)	<0.0001 *
Lympho (%) ^b^	3 (0.8–4)	3 (1.6–4)	0.77
Eosino (%) ^c^	0.2 (0–8)	0.6 (0–3.5)	0.84
Macrophage (%) ^b^	30 (18.3–61.5)	92.5 (85–96)	<0.0001 *

Data are expressed as medians (interquartile range) or numbers. TCC: total cell counts. ^a^: Within n = 8, after n = 11; ^b^: within n = 9, after n = 11; ^c^: within n = 7, after n = 11. * *p* < 0.05.

## Data Availability

The quantitative datasets that support the conclusions of this article are included in the article. More detailed datasets are available from the corresponding author upon request.
